# “Was that a success or not a success?”: a qualitative study of health professionals’ perspectives on support for people with long-term conditions

**DOI:** 10.1186/s12875-017-0611-7

**Published:** 2017-03-20

**Authors:** John Owens, Vikki A. Entwistle, Alan Cribb, Zoë C. Skea, Simon Christmas, Heather Morgan, Ian S. Watt

**Affiliations:** 1 Centre for Public Policy Research, King’s College London, Waterloo Bridge Wing, Franklin-Wilkins Building, Waterloo Rd, London, UK SE1 9NH; 2 Health Services Research Unit, School of Medicine, Medical Sciences and Nutrition, University of Aberdeen, Health Sciences Building, Foresterhill, Aberdeen, UK AB25 2ZD; 3 Department of Health Sciences, University of York, Seebohm Rowntree Building, Heslington, York, UK YO10 5DD

**Keywords:** Diabetes, Chronic conditions, Parkinson’s disease, Quality of healthcare, Self-management, Outcome assessment

## Abstract

**Background:**

Support for self-management (SSM) is a prominent strand of health policy internationally, particularly for primary care. It is often discussed and evaluated in terms of patients’ knowledge, skills and confidence, health-related behaviours, disease control or risk reduction, and service use and costs. However, these goals are limited, both as guides to professional practice and as indicators of its quality. In order to better understand what it means to support self-management well, we examined health professionals’ views of success in their work with people with long-term conditions. This study formed part of a broader project to develop a conceptual account of SSM that can reflect and promote good practice.

**Methods:**

Semi-structured individual interviews (*n* = 26) and subsequent group discussions (*n* = 5 groups, 30 participants) with diverse health professionals working with people with diabetes and/or Parkinson’s disease in NHS services in London, northern England or Scotland. The interviews explored examples of more and less successful work, ways of defining success, and ideas about what facilitates success in practice. Subsequent group discussions considered the practical implications of different accounts of SSM. Interviews and group discussions were audio-recorded, transcribed and analysed thematically.

**Results:**

Participants identified a wide range of interlinked aspects or elements of success relating to: health, wellbeing and quality of life; how well people (can) manage; and professional-patient relationships. They also mentioned a number of considerations that have important implications for assessing the quality of their own performance. These considerations in part reflect variations in what matters and what is realistically achievable for particular people, in particular situations and at particular times, as well as the complexity of questions of attribution.

**Conclusions:**

A nuanced assessment of the quality of support for self-management requires attention to the responsiveness of professional practice to a wide, complex range of personal and situational states, as well as actions and interactions over time. A narrow focus on particular indicators can lead to insensitive or even perverse judgements and perhaps counterproductive effects. More open, critical discussions about both success and the assessment of quality are needed to facilitate good professional practice and service improvement initiatives.

**Electronic supplementary material:**

The online version of this article (doi:10.1186/s12875-017-0611-7) contains supplementary material, which is available to authorized users.

## Background

“Support for self-management” (SSM) is a prominent strand of health policy in many countries, particularly for primary care. In the context of the rising prevalence of long-term conditions, encouraging and enabling people to do what they can to manage their own health issues is seen as necessary both to limit reliance on professionally delivered interventions and to render effective health service provision more sustainable [1–4]. SSM can also be presented as an important contributor to people’s overall wellbeing, and as a feature of ‘person-centred’ service provision, for example by being respectful of and responsive to diverse individuals, and by helping to meet expectations for participation and autonomy [5–10].

These multiple ambitions are not always fully or equally reflected in the evaluation of initiatives to promote SSM or in assessments of professional practice or service quality. Beyond the attention that is paid to service usage and costs, there is a strong tendency in such assessments to focus on biomedical indicators of health or risk and on the behaviours (or psychological predictors of behaviours) that can contribute to these. For example: the UK Quality and Outcomes framework has emphasised blood pressure and blood glucose levels, and there is currently much interest in the Patient Activation Measure which assesses people’s knowledge, skills and confidence in relation to professionally recommended condition-management activities [11–13].

Of course, any indicator is likely to present a simplified picture of the quality of professional service provision, but it is important to consider which aspects of practice and quality they emphasise and encourage, and which they might obscure and hinder. To better understand what it means to support self-management (and well), and to develop a richer understanding of the purpose and quality of professional support for patients with long-term conditions, we investigated and engaged critically with health professionals’ views of what counts as success in their work supporting people with diabetes and/or Parkinson’s disease. The investigation was part of a wider project that sought to develop the conceptual underpinnings of health and social care support for people living with long-term conditions. This project built on work that had previously identified some important concerns about prevailing conceptions of collaborative and patient-centred care in the context of chronic illness [5, 14]. It used a combination of a literature review [15], interpretivist qualitative research with health professionals, applied philosophy (drawing in particular on the framework provided by the capabilities approach (see: [16–19]) and knowledge exchange events with a range of stakeholders. The ambition was to develop a refreshed conceptual account of SSM that could help recognise and promote the kinds of person-centred approaches to support that (a) respond to what matters to patients and in ways that they value (see, for example: [20–22]), (b) health professionals themselves recognise as good, and (c) reflect philosophically defensible assumptions about patients as people living in diverse social and material circumstances.

## Methods

This paper draws on the qualitative research (individual interviews and subsequent group discussions) with health professionals. For these, we purposively focused on diabetes and Parkinson’s disease as two (clusters of) long-term conditions that between them could help illuminate the diverse considerations salient for more general discussions about SSM. Diabetes and Parkinson’s are both increasingly prevalent chronic conditions for which long-term support is often delivered by a range of professionals in primary and secondary care. Both will deteriorate over time and both can co-occur with other long-term conditions. They differ, however, in terms of: the kinds of impacts that they (and treatment and self-management regimes) have on patients’ lives; the extent to which patients’ behaviours can influence biomedical disease control; and the extent to which professional practice relating to them is the subject of guidelines, quality assessment and health service reward systems.

### Individual interviews

#### Recruitment, sampling and consent

We used a combination of publicly available information (staff listings available online) and personal contacts to identify a diverse sample of health professionals who worked with people with diabetes and/or Parkinson’s disease in several NHS jurisdictions in each of three broad regions/countries (London, northern England and Scotland). We kept a summary log of participant characteristics (profession, geographic area, practice setting, focus on diabetes and/or Parkinson’s Disease, and gender) to monitor diversity in our accumulating sample and inform our recruitment efforts. The sample was developed in part through a “snowball” approach, and recruitment of later interviews was targeted to address important gaps in the diversity profile of earlier interviews (see Table 1). Professionals initially received a participant information leaflet which presented the aim of the study as ‘to develop better ways of thinking about helping people to manage and live well with long term conditions’, and invited them to take part in an audio-recorded interview about their experiences of working with people with diabetes or Parkinson’s disease. The potential interviewer (JO or ZS) contacted those who expressed interest to answer any questions and if appropriate arrange an interview. All participants gave written consent before being interviewed.

**Table 1:** Sample characteristics

Participants in individual interviews (*N* = 26)			
*Professional backgrounds*	*N*	*Gender*	*N*
General practitioners	4	Male	11
Practice nurses	2	Female	15
Nurse specialists – diabetes	5		
Nurse specialists – Parkinson’s	2	*Location of practice*	*N*
Support worker	1	London	8
Dietician	1	North of England	6
Physiotherapist	1	Scotland	12
Clinical psychologist	1		
Medical specialist – diabetes	1		
Medical specialist – elderly care	1		
Medical specialist – neurology	6		
Medical specialist – psychiatry	1		
Participants in Group discussions (*N* = 30)			N
Regional group of (medical) specialists	Parkinson’s	Scotland	11
Regional group of nurses/allied health professionals	Diabetes	Northern England	6
Regional group of nurses/allied health professionals	Parkinson’s	Scotland	3
Mixed professional group, working in same area	Diabetes	Scotland	7
Mixed professional group, working in same service	Diabetes	London	3

*Note*: 5 professionals who took part in individual interviews also participated in a discussion group

### Data generation

Most interviews were conducted face to face, in NHS or University offices, but five were conducted by telephone due to participant rescheduling and/or transport challenges. The interviews were semi-structured and conducted in a non-judgemental, conversational style by one of two interviewers (JO, ZS) whom participants knew were non-clinical and interested primarily in understanding health professionals’ views about their work supporting patients to self-manage chronic conditions. The interviews lasted 20-100 min with most in the region of 45 min.

The interviews were supported by a topic guide (See Additional file 1) to ensure coverage of key issues relating to the overall aim of understanding health professionals’ views of what successful support for patient self-management looked like and what, in their experience, helped and/or hindered this. Our focus on success was informed by the literature review conducted earlier in the project, which identified an important distinction between supporting people to manage their condition(s) well and supporting them to manage well with their condition(s), and noted a lack of critical attention to questions about the purpose of SSM [15]. The first two interviews were conducted as pilots (participants agreed to give feedback on the questions and approach, as well as to be included in the sample), and informed a decision to continue using the topic guide as prepared.

We started the interviews by inviting participants to give a brief description of their current role and how they had come to it. We then asked for examples from their practice that for them represented greater or lesser degrees of success (and shifts between these) in their work with people with diabetes or Parkinson’s. We also invited them to: reflect on how they were defining success; consider how patients’ views about success might compare with their own; tell us what helped and what hindered more consistent success in practice; and comment on what they understood and thought of policies promoting ‘collaborative’ working with patients. Particular care was taken to frame questions about “success” in ways that would elicit the professionals’ own personal constructs, and avoid (in so far as was possible) imposing particular ways of thinking. For example, we avoided asking about ‘quality’, ‘effectiveness’ or ‘outcomes’ and asked health professionals to describe occasions when, in their view, their work supporting patients to self-manage had gone well and times when it had gone not so well.

### Group discussions

The discussion groups were conducted after the preliminary analysis of the interview data (see Data Analysis, below), and were designed to refine our emerging ideas about conceptualising SSM.

### Recruitment, sampling and consent

Participants were recruited from the same broad regions and NHS jurisdictions as for the individual interviews. Information leaflets were distributed by interested professionals in leadership roles. These leaflets requested participation in an audio-recorded discussion with other professionals that would include constructive critical comment on the research team’s ongoing work on ways of thinking about how health and social care staff help people to manage and live well with long-term conditions. Five groups (Table 1) met on NHS or university premises. Participants gave written consent prior to the recording of their discussion.

### Data generation

We developed stimulus materials based on three main sources: our prior literature review [15]; insights and examples from our preliminary analysis of the data from individual interviews with health professionals; and our ongoing reflection, which was informed in part by the philosophical theory mentioned earlier and the broad notion that SSM should be oriented towards helping people with long-term conditions live (and die) well with those conditions [23]. The stimulus materials comprised two summary descriptions of contrasting perspectives on SSM and six written vignettes describing people with type 2 diabetes or Parkinson’s disease whose condition-management was less than ideal by conventional biomedical standards, and who might present a challenge for health professionals working to support them. Group discussions were conducted by JO and AC in London, IW and VE in northern England, and VE and ZS in Scotland. After initial introductions, we asked participants to consider how they thought professionals who adopted the two contrasting perspectives on SSM would work with the person described in one of the vignettes and (among other things) how they would judge their success. Following a short individual reflection exercise, we facilitated a more open discussion about the relative merits of the perspectives presented in the two accounts. Researchers made brief field notes after the sessions.

### Data analysis

The interviews and group discussions were audio-recorded and transcribed verbatim. Data management and preliminary analysis was facilitated by the use of NVivo 10 text management software. We developed an initial coding framework for the interview data to reflect both our research questions and key insights noted during team discussions of six interview transcripts. The initial coding framework was applied by JO and ZS. Further analysis for this paper was conducted by JO, VE and AC, and agreed by the wider team. It focused first on data coded as responses to the requests for examples of more and less successful work, questions about professionals’ definitions and patients’ perspectives on success, questions about what helped or hindered more successful working, and expressions of positive and negative emotion. Discussion group transcripts were read separately and data interpreted as relating to ‘success’ was coded manually. VE re-read all the transcripts as wholes as an interpretive check before we finalised our analysis of the range of aspects and elements of success and of the kinds of consideration that health professionals brought to bear when assessing success or judging quality in particular contexts.

## Results

Sixty-five professionals were approached to secure 26 interviews. 5 subsequent discussion groups were held in which 30 health professionals participated (of which 5 had previously participated in individual interviews). Participants have been assigned pseudonyms here to preserve their anonymity.

In individual interviews, when asked to give examples of successful work with people with diabetes or Parkinson’s disease, some participants hesitated or commented that it was a difficult question to answer. None seemed to have a neat or well-rehearsed definition of success to produce on demand, but all could describe cases they associated with greater and lesser success, and all identified and discussed various (often inter-connected) criteria for success. Participants generally developed their comments about success as the interview progressed and they thought of additional examples and considerations. Although the depth of their reflections varied, all constructed success in multi-faceted and context-sensitive ways, and suggested important but far from simple links between aspects of success and judgements of quality.

The group discussions also recognised a number of different aspects and elements of success, suggested various conceptual and causal connections between these, and raised questions about whether and how particular aspects and elements should feature in assessments of the quality of professional practice.

To help manage the complexity within the accounts we first present the aspects and elements of success that were invoked or indicated, and then examine the various considerations professionals raised that have implications for assessments of practice quality.

### Aspects and elements of success

We have organised the aspects and elements of success into three broad and partially overlapping groups. Those in the first group (‘health, wellbeing and quality of life’) reflect what are widely understood to be the guiding purposes of practice and might be considered the primary or more ultimate indicators of success. Those in the second and third groups (‘what patients (can) do’ and ‘relationships and communication’) are sometimes regarded as mediating processes or intermediate indicators, although some are arguably also valuable in their own right.

To counter the risk that separating out the various aspects and elements of success will obscure the ways that they typically occurred alongside one another in participants’ talk, Table 2 illustrates some of the composite statements participants used to summarise their thoughts about success, and Tables 3 and 4 present slightly fuller excerpts as they featured over the course of two somewhat contrasting interviews. The quotation in Table 5 illustrates recognition that multi-morbidity could extend or complicate considerations in all three of the groups of aspects and elements of success that we have identified.

**Table 2:** Quotations illustrating the plurality of ideas about success

Quotation	Source
The ideal success is someone you have a good relationship with, who at the same time is well, is ticking all the boxes for excellence in biomedical control, and taking full responsibility, and keeping themselves well and healthy	Philip, General practitioner, northern England
I think if the person feels more in control and happier. I probably should say more that they’re getting better HbA1c and hitting more targets… to get the actual QOF with this being a GP practice. But my initial desire is to make the patient feel better and they’ve got control over things and it’s their condition, they’re managing it.	Pippa, Practice nurse, northern England
Somebody that maybe needs input from me less than they did originally… that would be a success. Success would be somebody that believes, that they can actually feel more confident to manage their diabetes, it’s important in their lives, and they can walk out of there knowing when they need to ring me… And I think from a sort of medical perspective… from a biochemical perspective, has the HbA1c been reduced, have the cholesterol and blood pressure reduced?… Because some people believe they’re doing amazingly well, but maybe they’re not… So I think that will be the three things: less contact; they’re feeling well in themselves (better in themselves), and the biochemical changes.	Shania, nurse specialist, diabetes, London
I - So in terms of what success looks like for your team, how do you tend to evaluate that?	Mixed discussion group, diabetes, London
P - That’s an interesting question. Our commissioners are very focused on HbA1c so they’re very much focused on biomedical outcomes and… because that translates into money… so that tends to be what we’re judged against in the main. But we also obviously would measure things like psychological functioning, social support and psychiatric morbidity as well, and where - we’re currently in the process of doing an evaluation of the service which would include all of those measure and we’re going to just… I guess come up with a quality of life index as well, so we’re working with our health colleagues in doing that. So we’re being forced to look at the biomedical side of things, the service use but our own focus would be to allow people to engage better with their health care that they can be independent, healthy people in the future, who don’t end up in hospital.

**Table 3:** Talking about success (a) Suzanne, specialist nurse, diabetes, London

Excerpts are presented in the order they arose in the interview	
Interviewer:	… can I ask for some examples or case histories from your experience, to illustrate what your idea of success might look like?
Participant:	Oh, gosh, right, yeah. So I mean… I guess that can vary enormously from the type of clinics that we’re doing. So for instance, in an antenatal clinic… success is a healthy live baby and healthy mum… And then, of course… somebody with a chronic condition where you’re just supporting them living with the condition… We, of course, as health professionals, want someone to have as best HbA1C to reduce the risk of complications in the long term, as well as to be able to live a happy, healthy life, as it were… The people we get are more and more complex… We’re never going to achieve the ideal HbA1C for everyone… So if we can even just chip away and support them to live better with their diabetes we’re hopefully doing something to support them in a positive way…
Interviewer:	… Can you think of some examples, again from your own experience, to illustrate what an unsuccessful partnership with a patient might look like?
Participant:	I think one where there’s no connection, or where the patient probably isn’t at the right place to have a discussion about managing their diabetes, for whatever reason… We do have consultations where we think “Oh that didn’t go very well”… when you feel like you’re not getting very far with someone for whatever reason
Interviewer:	… So thinking about the things you’ve said… can we think about how we might define the concept of success?
Participant:	Gosh, yeah, that is so hard, isn’t it? Because the concept of success, I suppose, is about… the people with diabetes that had long term outcomes, the effect on the NHS, all those sort of things … What you really want to achieve is to be able to support someone to self-manage their diabetes so that they do not get (or they reduce the risk of getting) long term complications… And that’s success. But success on a day-to-day basis is about chipping away, and having a long term - and motivating people to take some action about their diabetes…
Interviewer:	… Do you think patients would agree with how we’re defining success here?
Participant:	Well no, not necessarily. Because success – another success could be for instance [with someone who is] having a really hard time with their blood glucose swinging all over the place [that] they have much more stable blood glucose levels that enables them to feel more confident about living their life without the risks of feeling unwell in the morning because their blood sugars are high, or having hypos in the middle of work situations, which are incredibly embarrassing… So that would be incredible success for an individual. That would be success for us, too, but then we’re always wanting more, aren’t we… for the long-term risks of complications?

**Table 4:** Talking about success (b): Craig, Medical specialist, neurology, Scotland

Interviewer:	… examples or case histories from your experience of something that would illustrate your idea of success?
Participant:	Erm. I guess that depends on what we’re doing and it also depends what stage of the condition the patient’s at. So … for Parkinson’s I would say that there are four stages of the condition … So what counts as a successful encounter… depends on what the issue is for that patient at that stage, and my guess is that you might define success and failure differently for different scenarios. Although I dare say there will be some features that would be common to all.
Interviewer:	And can you say a bit more about what they might be, from your experience?
Participant:	Erm. I suppose, if you’re looking at generic things, I suppose it would be issues with communication: honesty, accuracy … building and maintaining a relationship. From a patient’s point of view I think they value seeing someone who *knows* about their condition … I suppose a successful consultation has to have sufficient time, but it also has to occur at the right time… Those are the things that spring to mind. …
Interviewer:	… do you have any examples … of what you would describe as maybe a successful early encounter?…
Participant:	I think you would have to ask the patients about that, you know. What is success from my point of view might be rather different from success from their point of view… One encounter that I recall… was a… worker in his 40s with a bit of a tremor, and I told him I thought he had Parkinson’s Disease. He didn’t like that [and went off and saw a neurologist elsewhere who] said it was probably a form of essential tremor, so he was very happy… Unfortunately his symptoms got gradually worse [and the other neurologist] eventually agreed that he did have Parkinson’s Disease… So now, I don’t know, was that first encounter a good one or a bad one? I was right, and I love being right [laughter]… but I told him information that he wasn’t happy with, and which maybe he wasn’t ready to accept at that stage. So I don’t know how you judge whether that was a good encounter or not…
Interviewer:	… would you be able to define success in your view of encounters with people with Parkinson’s [in the early stages]?…
	Yeah, so communication, I suppose accuracy in the information that we provide… the way in which it’s communicated, ‘cause I guess the quality of communication will always make a difference to how people take things in. And the back-up available. I suppose that’s the other thing… quality … isn’t necessarily [just] about what happens during that appointment … For example, if somebody is complaining that their speech has deteriorated and they want a drug to make that better, if I say “Well, no drug is going to make your speech better…, but we’ll refer you to a speech therapist”, they may be disappointed but they are at least going to see someone who can maybe advise them about that symptom and help them cope with it better…
	… In a sense, I don’t have a desired result really, other than the best for the patient. It has to be the patient’s desired result really, not mine.

**Table 5:** Managing multiple conditions

*Multi-morbidity could extend or complicate assessments of success in all three broad categories of aspects and elements of success. These comments are from an interview with Lucy, clinical psychologist, diabetes, London.*
For example, someone I saw with diabetes and heart disease… So he was, I suppose, quite suicidal really when I saw him initially, and by the time we’d finished much more assertive, confident and the suicidal ideation had diminished, able to have constructive relationships with family members, so that was a good outcome. However, the medical conditions sort of were either maintained or slightly deteriorated, so there wasn’t really progress on that front…
I supposed there’s staged outcomes that we see sometimes as – sometimes you’re treating the depression and anxiety first before they’re in a position to feel motivated enough to then self-manage appropriately…. And the next stage might be to form a healthier relationship with their health team, so just thinking about the rapport they have and how confident they feel about asking questions in consultations, so you’re kind of facilitating that bit and *then* they might be in a position to start addressing their long term condition in a more helpful way…
Another factor [relevant to success] is patient co-morbidities because that comes up a lot. So they’re roughly juggling more than one long-term condition. And so if that other long term condition starts to deteriorate it impacts on their – but even ability to come to appointments regularly, and the number of medications people have to take, and not understanding what’s for what. I’m really passionate about the idea that often people need some kind of co-ordinator figure in order for them to self-manage well and that’s not – we haven’t quite got there yet in health care systems… I mean there’s a lack of joined up thinking across long term conditions and that’s not compatible with the reality which is that patients are juggling long term conditions - that’s often a barrier to successful outcomes.

#### Success in terms of health, well-being and quality of life

Not surprisingly, ideas associated with patients’ health, wellbeing or quality of life featured somehow in all participants’ comments about success. Some discussions, particularly those in which participants considered the ways performance was assessed in the organisations in which they worked, focused on biomedical markers, the presence or risk of symptoms of disease, or functional limitations. All participants made some reference, however, to patients’ broader health, emotional and psychological wellbeing and/or quality of life, and some foregrounded and emphasised these broader notions. We illustrate this range here.

- *Biomedical markers*
  Biomedical markers featured particularly in the interviews relating to diabetes. For example:
  One of the main parts of our role, from the diabetes nurse perspective, is trying to improve patient’s glycaemic control… but the role is widening, because we do local blood pressure and cholesterol, so [success is] trying to reduce the risk factors for later on complications. (Shania, nurse specialist, diabetes, London)
  Mentions of biomedical markers were usually nested within broader ideas about future risk and/or people’s wellbeing. This nesting was particularly evident in discussion groups when participants discussed alternative perspectives on the purpose of support for people with long-term conditions.
- *Symptom control*
  ‘Adequate symptom management’ and the prevention of symptomatic decline or complications were widely referred to as key aspects of success. For example:
  I suppose the kind of medical agenda in coming into encounters with people with Parkinson’s Disease is very much to improve their symptoms and to make them less stiff and slow. (Matthew, medical specialist, elderly care, Scotland)
  I think symptom control would be a success, like if someone is coming in with significant symptoms around their diabetes, like if they’re getting up at night to pee. If we can reduce that, they come in saying, “Oh I’m not weeing as much at night; I’m not as tired.” So symptom control - people do notice that one (Maureen, dietician, London)
- *Getting on with daily life*
  Symptom control was often connected with a broader interest in people being able to get on reasonably independently with their daily lives and to live as ‘normally’ as possible. Some participants emphasised these broader concerns as they discussed success. They sought to help people to manage and maintain their valued activities, roles, settings and positions, and described success in terms of being ‘able to keep them at home or keep them in the kind of care environment that they want to be kept in’ (Angela, nurse specialist, Parkinson’s disease, Scotland) and
  ‘keeping people… in the lifestyle that maximises their quality of life. Which sounds all a bit nebulous but basically if they are working you want them to try and carry on working, if they are retired and have hobbies you want to be trying to the keep them enjoying life to the best of their ability, in the knowledge that this is a progressive disease. (Alistair, medical specialist, neurology, Scotland)
- *Psychological wellbeing*
  Emotional wellbeing and psychosocial coping, including the ways people related to their condition, were also recognised as salient for success, both in their own right and for facilitating condition-management and participation in social activities that could otherwise be important. For example:
  One of my favourite success stories was … a lady who was … very intelligent and … seemingly in control of her life, but … she put her hands in a house roof shape over her head and she said, “I feel like diabetes is always over me all the time”. And after a few weeks I said to her … “What’s your diabetes feeling like now?” and she said, “I feel like I’ve got a little badge on my lapel saying, ‘I’ve got diabetes’ but it’s just a little badge, it’s not over the whole of me”. (Kate, nurse specialist, diabetes, northern England)
  And, as an example of a *less* successful case:
  … a person who is really well. He’s had [Parkinson’s disease] for 11 years, he can punch a punch ball, he can ride a bike… but he’s absolutely psychologically wound up by his tremor on a daily basis, … it just embarrasses him, he can’t cope with it (Christina, medical specialist, neurology, London)
- *General health and happiness*
  Some health professionals foregrounded broader concepts of health, happiness, well-being or quality of life, stating, for instance, that success is “sort of about health and happiness outcomes really” (Kate, nurse specialist, diabetes, northern England), that “a happy patient is a successful thing” (Isobel, general practitioner, Scotland), and that they could consider themselves successful if they had helped people to “cope better and feel better” (Angela, nurse specialist, Parkinson’s disease, Scotland).
- *Avoidance of undue treatment burden*
  Several health professionals also factored into their considerations of success the need to ensure that health care practices did not themselves undermine people’s sense of wellbeing or their broader quality of life. For example:
  Success has got to be much more about making the patient feel they can cope with the help that health carers give them, that it’s not an ordeal coming to the clinic, that we’re not rubbing their face in their mortality every time we discuss their condition… perhaps improving outcomes, but even not improving outcomes as long as you don’t make them feel even more miserable and oppressed by this condition that they have to live with, so that’s a success enough for me (Jeremy, medical specialist, diabetes, Scotland)

#### Success in terms of how well people (can) manage

Consistent with the general idea that professional support should help people to ‘self-manage’, success was widely associated with interlinked ideas about: what people did or were enabled to do for themselves to manage (and manage well with) their long-term health conditions in their daily lives; supporting people’s motivation and confidence to self-manage; people contributing to problem solving and goal-setting in consultations; and a reduction in unwanted or unnecessary dependence on professional services.

- *People being able (and professionals enabling them) to deal effectively with their health conditions in their daily lives*
  Not surprisingly, a number of examples and definitions of success related somehow to people’s abilities to get on with at least the basics of condition management (e.g. taking medication and making situational adjustments to doses). Professional facilitation of more effective self-management also featured in considerations of success. For example:
  I think successful’s where someone is taking ownership and getting involved in their own management of the condition. I think successful as well is, when problems arise, addressing provision of support, so having a responsiveness in the system and myself, of trying to respond to needs as they arise. (Sean, medical specialist, neurology, Scotland)
  People’s abilities to manage their conditions were generally seen as important, either as causally related to, or partly constitutive of, various aspects of health, wellbeing and quality of life. Broader ideas about people being able to manage or cope well with their long-term conditions also featured as important aspects of success in individual interviews, and the idea that the purpose of SSM includes enabling people to live well with their (often multiple) long-term conditions was strongly endorsed in group discussions. Examples of these overlap with examples of “Getting on with daily life” and “Psychological wellbeing” (described above).
- *People contributing to problem solving and goal setting, including in consultations*
  Considerations of what people were able and enabled to do within the contexts of health care provision also featured among ideas about success.
  I: Can I ask for some examples of case histories from your experience to illustrate what your idea of success might look like in diabetes?
  P: Well I think the idea is, is when the patient actually comes up with their idea of how they can make things better rather than me dictating to them that “You need to make this change, you have to do this” … When we put that [patient’s idea] into our plan, they then come back the next time and they’re very proud of themselves because… they’ve done it themselves, so I find that a great success’. (Dorothy, nurse specialist, diabetes, London)
- *People not being ‘unduly’ or long-term dependent on professional services*
  The avoidance or movement away from forms of dependence on professional services that either patient, professional or both considered unwanted or unnecessary were also identified as indicative of success, perhaps especially by professionals who specialised in working with people with particular difficulties managing (with) their conditions.
  I - So going back to the idea of success, what I’m hearing you saying is that success is partly having services that are accessible but it’s partly people becoming not too reliant -
  P1 - Yes, about non-reliance....because we know having to live with a condition like diabetes, at different time points people are going to find that really difficult and you know, people can reach burn-out at different stages and knowing that they can access support when they might need it but not that it’s an ongoing thing that they can rely on the whole time.
  (Group discussion, Diabetes, London)
  Several participants commented on the challenges of balancing the provision of support with the encouragement of ‘self-management’, and for some, striking an appropriate balance could itself be considered a success:
  I would see success as getting that right balance where the patient and the family feel they have got some control but they also know that they have got support when they need it, (Alistair, medical specialist, neurology, Scotland)

#### Success in terms of professional-patient relationships

When talking about success, health professionals also referred, with varying degrees of emphasis, to aspects of their relationships with patients. Several stressed the importance of establishing and maintaining trusting and durable relationships, and one described good relationships as “the bedrock of success” (Philip, general practitioner, northern England). We identified four broad kinds of reason for associating positive relationships with success.

- *Facilitating open and effective communication from patients*
  Good relationships were seen as important for encouraging patients to communicate and engage with professionals in ways that would in turn facilitate the provision of effective and appropriate support (e.g. to be honest about their symptoms, actions and “what their expectations are, what they feel able to change, what they need support to change and what they feel can’t be changed” (Philip, general practitioner, northern England).
- *Facilitating open and effective communication from professionals*
  Some professionals noted that positive relationships enabled them to raise difficult but important issues constructively and without undermining the person or their willingness to engage with health care. For example:
  Hopefully I have spent time investing in some sort of relationship with the patient and actually they know that I'm looking out for them, that I’m not just somebody that nags someone because that’s my job: I’m a doctor… You have to reach some sort of level of relationship with the patient before you can start to broach things in a sensitive way that they will accept (Paul, general practitioner, Scotland)
- *Keeping health care accessible*
  A recognition that people’s changing life circumstances as well as their changing (and often multiple) health conditions could impact their scope to act and ability to cope meant professionals were keen to ensure people felt able to ‘come back’ to services even if they had previously been unwilling or unable to follow recommendations. Good relationships were seen as important to facilitate this.
- *Constituting support*
  Although professional-patient relationships are often talked about in ways that present their value as instrumental, several comments indicated that they can also be understood as intrinsically important components of success, not least because positive relationships can contribute more as well as less directly to people feeling valued and supported. For instance:
  I think success is when a family thank you… that elderly couple who I still haven’t really solved their problem but she says, “I’m so grateful that you’re there to speak to me”… Sometimes… it’s someone who understands what they’re going through and sometimes they just want to talk to relieve the tension… people are grateful that you’re there to listen… (Angela, nurse specialist, Parkinson’s disease, Scotland).
  It’s more about keeping people engaged with you so that if they run into difficulties then they will trust you not to force our agenda on them… so it’s more about trying to help people kind of maintain their personhood in the face of an illness rather than kind of me regarding them as a person with an illness and treating it (Matthew, medical specialist, elderly care, Scotland),

### Assessing success and the quality of practice

Participants’ views about the relevance of different aspects and elements of success could, understandably, vary depending on the envisaged context and reason for assessing success. Our participants’ comments apparently reflected ideas about success that were responsive to shifting contexts and salient concerns. The aspects and elements the health professionals considered useful when engaging in supportive communication with particular patients could differ, for example, from those they considered useful for critical reflections on their own professional practice. In this section, we focus on how health professionals’ comments reflected and raised questions about assessing success. More specifically, we consider whether, why and how the various aspects and elements they associated with success could be appropriately used as indicators of the quality of their practice.

The key considerations can be summarised as relating to: the variability and contestability of both what matters and what is realistically achievable for particular people, in particular situations and at particular times; and questions about the appropriateness of assigning responsibility to health professionals (and, indeed, to patients) both in terms of prospectively allocating roles and tasks, and retrospectively holding to account and apportioning credit or blame).

Our participants recognised that their own perspectives on what matters and what is realistically achievable could differ from those of their patients, colleagues, managers or professional organisations. All gave some priority to the expressed views of particular patients, but the ways and extent to which they were inclined to do this, and the emphasis they gave to other considerations, varied. Participants also recognised that their remit or scope to make a difference was often somewhat constrained, and that patients’ experiences and outcomes did not always or only reflect the quality of their professional work.

These considerations were variously reflected in concerns about focusing too reductively on any one aspect or element of success, as we illustrate here.

### Concerns about a focus on biomedical or other markers of health

Our participants were clearly committed to supporting people to achieve potential improvements in their health, but had various reasons for regarding biomedical indicators or other markers of health as limited, inappropriate or not always relevant for assessing the quality of their practice. The main considerations were:

- *Biomedical markers are generally limited as indicators of health or wellbeing*
  As noted above, participants generally considered blood pressure, blood glucose etc. as readily measurable but imperfect proxies for important health states or risks.
- *The relevance of biomedical and other health markers and targets varies*
  Markers and targets that might be broadly relevant for some people could be inappropriate for others – particularly those with more advanced disease, multiple morbidities or otherwise complex problems
  In the diabetes population… they don’t necessarily need to lose weight. Some have come in to help with their weight, but some have been losing weight because their diabetes has been poorly controlled. … So some clinical measures aren’t helpful. (Maureen, dietician, London).
  Some participants also stressed that assessments of success should be guided by each person’s particular health-related values and priorities.
  I suppose I’m not in any position to tell them whether being stiff and slow and unable to walk is worse than feeling sick all the time or whatever their perceived side effect is (Matthew, medical specialist, elderly care, Scotland)
  A few suggested that if a service is oriented to support people to achieve the outcomes that matter to them, those personal outcome priorities should somehow be reflected in assessments of its success:
  When … we talk about what might Bert [a character in a vignette] want, the point is we’ve got to completely change the accounting system so then Bert has to make up the questionnaire as to which things get [quality] points, and Bert has to say how well they were achieved (Medical specialist, diabetes group, Scotland)
- *Biomedical markers do not reliably reflect (professional support for) patients’ self-management actions*
  Participants noted that even rigorous adherence to the behaviours that research evidence suggests are most effective for risk reduction will not guarantee achievement of biomedical targets or avoidance of deteriorations in health. These things are not fully under people’s control and cannot be reliably attributed to patients’, let alone health professionals’ behaviours.
  For example:
  People can make the life changes or do the right things or take the tablets at the right time, but the nature of the condition – and that can be diabetes or Parkinson’s – can mean they can deteriorate or their diabetic controls are off or the Parkinson’s gets worse despite everything we do. (Paul, general practitioner, Scotland)
- *A strong focus on standardised biomedical targets can be harmful*
  Especially in relation to diabetes, some participants were quite strongly concerned about performance-related finance systems that focused on standardised biomedical targets. The targets were seen as unrealistic for many people:
  Somebody with an HbA1c 80 plus isn’t going to be able to get an HbA1c in the 50s in a few months (Kate, nurse specialist, diabetes, northern England).
  But beyond this, some participants expressed concern that an explicit emphasis on targets in clinical practice could counterproductively lead patients to become demotivated, self-critical and distressed in the face of persistent ‘failure’. Several participants were also concerned that the systematisation around standardised targets had negative implications for how health professionals felt they had to work and how the quality of their work would be judged. They lamented, for example, a culture of box ticking, shifting performance indicators and feeling “totally frustrated and suppressed and oppressed by the targets” (Jeremy, medical specialist, diabetes, Scotland).
  Several also expressed concern that targets could undermine efforts to work collaboratively and sensitively with each particular person and their circumstances:
  I never thought targets was a very good idea in the first place I think because that prevents you … I suppose it prevents to some extent treating people as individuals you know you’re just trying to get them down within their target level without necessarily taking into account their personal circumstances, their personal problems, you know other issues that may play a part. (Mark, general practitioner, northern England)

### Concerns about patients’ behaviours as indicators of good quality practice

Similarly, although participants sought to encourage and enable people to maintain or adopt behaviours likely to benefit their health or wellbeing, and could clearly welcome the willing adoption of these behaviours (as long as they did not become pathologically obsessive or otherwise disruptive of overall wellbeing), they had concerns about their use as indicators of the quality of professional practice. These concerns related mainly to considerations of fairness and attribution.

- *People’s behaviours can be constrained by various biomedical, psycho-social and material-financial factors*
  Participants were acutely aware that co-morbidities and other issues not readily within patients’ or health professionals’ control could render recommended behaviours unrealistic for some particular people (or at particular times).
  Here we’re in the poorer area … So [it’s all] very well for me to suggest that they eat their five fruits and veggies a day when, you know, in fact, they really can’t even afford to buy a bottle of milk. So you have to take all of those things into consideration. (Dorothy, nurse specialist, diabetes, London)
  Diseases are sometimes complex… People are living … in a normal life, so your intervention is only a small part of what is a very big picture, and … life throws shit in the works… so in the context of people’s health and their ability to do a lot of things, then actually sometimes this kind of intervention… sometimes falls by the wayside. (Stephanie, physiotherapist, London)
- *Health professionals cannot always take blame or claim credit for (changes in) patients’ behaviours*
  As with biomedical markers, behavioural changes that might be regarded as *a* success (or failure) could not necessarily be counted as a *professional* success (or failure). At the failure end of the spectrum, several participants mentioned people who for various reasons did not engage enough with services to give health professionals a chance to influence them, and there was also a widespread recognition that health professionals could not and should not control how people go about their daily lives. At the success end, some participants talked about people who had long seemed unable or unwilling to work on their health problems who suddenly started to do so. They emphasised that such turn-arounds were more likely to be due to a life event such as getting a new diagnosis, meeting a new partner, or finding a new job, than anything that health professionals might be doing differently. For example:
  She acknowledges that this information has been available to her for years… but she’s never really listened to it before and she’s kind of not wanted to know about it… I just happened to have come across her – or she’s been referred to me – at a good time where I can be very successful with her (Kate, nurse specialist, diabetes, northern England)

### Concerns about the use of patient satisfaction or happiness indicators

While patients’ happiness and health professionals’ respect for their expressed wishes were clearly important, none of our participants suggested that these should be used as a primary indicator of the quality of their practice. Indeed, several suggested that an overemphasis on happiness as an indicator of success could be problematic without simultaneous and situation-sensitive attention to people’s health and health-related behaviours. As one explained, it is “very easy to collude with patients” in ways that could contribute to temporary or superficial happiness but be detrimental to their understanding of their situation and/or their longer term health and wellbeing (Jeremy, medical specialist, diabetes, Scotland).

### Concerns about the limitations of a focus on professional-patient communication and relationships

In part because of the difficulties of attributing credit for success in other domains, some participants were inclined to evaluate their practice by focusing on their own actions and aspects of their relationships with patients, including their contributions to enabling people to live well despite their conditions. For example:

From a medical point of view, successful means that their Parkinson’s is well controlled, i.e. doesn’t really interfere with their life or lifestyle, for as long as possible… If I am being brutally honest, probably that is more to do with the type of Parkinson’s … than anything I do… There are different types of Parkinson’s in terms of the speed of progression, whether someone develops a dementia… and we don’t really have control over that… So I think from a success point of view, in terms of when I look at patients and say “Have I done a good job there?”… I would see it probably more holistically, so have I managed to establish a relationship with that person which has stood the test of time (because these people you will follow up for a number of years)? Have they found *me* helpful, in other words do they find the input and the support that they get from me and the service… has helped them deal with their Parkinson’s in as positive a way as possible without becoming over dependent… (Alistair, medical specialist, neurology, Scotland)

It was notable, however, that the quality of relationships was not seen as a sufficient indicator of quality overall. Although the interest in the quality of relationships in our sample was sometimes linked to a recognition that health care could make little difference to biomedical outcomes, this could not be interpreted as a sign of complacency. Participants continued to attend to other indicators of success (or failure), and particularly in cases where poor outcomes might in principle have been avoided, they asked themselves what more they could have done in the circumstances. In doing this, they were aware that appropriate action on their part included a need to respect as well as to support patients as moral agents, and this could include recognising that patients had some responsibility for what happened to them. For example:

His biomedical control has always been poor and he’s never really looked after his diabetes particularly well, but we have maintained a good relationship, he has always been happy to come and see us at regular intervals. He has always apologised for the fact that he doesn’t look after himself as well, and indicated that he would do, but also indicated that he was reasonably happy living the way he was living. But unfortunately in recent years, the fact that his diabetes has been poorly controlled has really caught up with him and he has now got all sorts of problems. And I guess, you know, on one level I feel that is a failure… on my part and the team’s part, but it is also a failure on the patient’s part as well. (Philip, general practitioner, northern England).

## Discussion

Our data indicate that health professionals working to support people with long-term conditions recognise success in their work in multiple overlapping ways. They mentioned various aspects and elements of success that seem to operate, and make sense, in complex inter-relationships with one another. For example: the value of biomedical indicators usually depended on their significance for less biomedical ideas about health and quality as well as length of life; psychological and holistic ideas such as wellbeing or quality of life were contrasted with but not viewed either in isolation from or simple opposition to biomedical ideas; and accounts of the importance of ‘what patients (can) do ’ and ‘good relationships’ were typically co-present but in overall discussions of success were not straightforwardly related either to each other or to health or quality of life.

Participants’ views of success in their work with people with long-term conditions were highly context-sensitive and in various ways patient-specific. The relevance they attached to particular kinds of states, actions and interactions (or changes in these over time) could depend on a variety of features of patients and their circumstances. These were not limited to co-morbidities: they included people’s social situations, personal priorities and capabilities. Some of the views expressed in this study are potentially applicable beyond long-term conditions, but we did not ask participants to offer a general view of good or high quality professional practice and they did not comment on how good SSM relates to good practice per se.

It is a strength of our study that our samples included participants with a variety of professional roles, levels of seniority and geographical locations, and focused on two contrasting chronic conditions that were deliberately chosen for their potential to illuminate different aspects of SSM. However, attention to conditions other than diabetes and Parkinson’s disease, and more detailed probing about issues relating to multi-morbidity and professionalism more generally might generate further insights. Moreover, the sample for the individual interviews was relatively small and participants by and large agreed to participate because of an existing interest in or concern about ideas and practices surrounding SSM. Some had clearly been reflecting carefully for a long time on the challenges of working collaboratively with people with long-term conditions, and our sample might be skewed towards the more reflective and/or ‘person centred’ end of the professional spectrum.

In writing up the results and the discussion we purposefully selected quotations that most clearly illustrated key themes. We have sought to represent the range of views, but we have included several quotations from some participants whose reflections added particular richness. We do not claim to provide a comprehensive account of success, but believe our data and analysis does open up considerations about the complex and multifaceted nature of successful SSM that have substantial relevance for policy and practice.

As far as we are aware, this exploration of what constitutes success in support for self-management is the first of its kind. As our earlier review suggested, the existence of different practical interpretations of concepts such as ‘patient empowerment’ and ‘patient involvement’ has been documented on a number of occasions, but questions about the purpose of support for self-management (or health care more generally) have been somewhat neglected [15]. The elasticity of ideas about ‘health’, ‘managing better’ and ‘quality of life’ allows for an easy appearance of consensus about purpose, but can obscure important differences that lead to varying views of success [23].

The plurality, inter-connectedness and context-sensitivity of the aspects and elements of success identified in this study not only illustrate the complexity of the term, they also indicate the need to hold multiple considerations together in both the practical pursuit and the assessment of success over time. Given that the different aspects and elements of success can sometimes be in tension with one another, health professionals face the challenge of finding situationally appropriate balances, and perhaps having to make trade-offs between them, as they work.

For these and other reasons, assessments of the quality of professional performance also require balancing a number of technical and ethical considerations. They demand attention to questions about attribution and fairness as well as questions about the appropriateness of different professional responses to uncertainties about what is possible and realistic.

Our findings and analysis chime with and lend support to a number of the various rationales for concerns that have been expressed previously about systematised (and especially incentivised) assessments of quality that rely on single or narrow sets of indicators (e.g. [24, 25]).

Our findings highlight particularly the limitations of quality assessments that are insufficiently sensitive and responsive to patients’ particular (and changing) situations and personal perspectives, and to the complex circumstances within which health professionals work (including multiple and potentially competing regulatory and ethical norms). There seems currently to be quite a disjunction between the range and complexity of (sometimes competing) considerations that health professionals draw on in their day-to-day assessments of success and the more restrictive outcome and quality monitoring frameworks that rely on indicators (typically biomedical markers and assessments of patients’ knowledge, skill, confidence and motivation).

The development of approaches to quality assessment that can adequately reflect the plurality, variability and contestability of what matters, what is realistically possible, and what is professionally appropriate is of course no simple matter [21]. It is not clear that any widely and readily applicable set of indicators could be devised that would reliably deliver assessments of the quality of professional support for people with long-term conditions; let alone that such indicators could be developed in ways that would command consensus as fair and accurate. Indicators that are responsive (and/or able to assess professional responsiveness) to each person and what matters to them are likely sometimes to miss issues that might reasonably be considered of general importance. A focus on aspects or elements of success that seem closer to what ultimately matters in terms of health, wellbeing and quality of life seems to mean a focus on aspects or elements over which health professionals have less control, and conversely a focus on assessing what health professionals have more control over can perhaps get less close to achievement of what matters to patients. Assumptions about what is realistic (which seem important for moderating judgements of professional practice) will be highly contested, and assessments of the kinds of circumstances patients and health professionals are operating in, and the resources they have at their disposal (similarly important for moderation) risk being intrusive and are likely to be unwieldy and difficult to interpret.

This is not an argument against doing anything to assess and try to improve the quality of professional support for people with long-term conditions, but it does suggest a need to look critically at how success is currently understood and measured within health systems, and probably to develop more sensitive and flexible methods for assessing quality that take account of the complex and situated nature of success. We suggest that such developmental work should attend to the range of views of health professionals and patients, and be guided by a combination of critical reflection and the broadening philosophical perspectives that have supported our own research. The health professionals we spoke to welcomed the opportunity to reflect on and discuss questions concerning successful SSM, and it may be that creating space for continued reflection has potential as a means of developing both the practice and evaluation of SSM.

There will continue to be a need for high levels of nuance and caution in attempts to evaluate (or appraise and interpret evaluations of) the quality of support for people with long- term conditions. Our analysis suggests that professional educators, service managers and policy leaders in particular need to operate with a rich and well-grounded picture of the multi-dimensional nature of successful SSM, and of the challenges that professionals face in working with people with long-term conditions, if they are to understand and support improvements in this. A nuanced assessment of the quality of SSM requires attention to the responsiveness of professional practice to a wide and complex range of personal and contextual states as well as actions and interactions over time.

## Conclusions

The grounded account of successful support for self-management that we have offered indicates a need for more explicit consideration of the complexity of judgements of success and quality in this domain.

If assessments of the quality of care are to be defensible and warrant the confidence of patients, health professionals and broader publics, attention should be paid to the plurality of aspects and elements of success, the features of particular patients and their (changing) situations that moderate the salience of these, and questions of what is realistic and what is fair in terms of the allocation of both prospective and retrospective ideas about responsibility.

## Additional file

Additional file 1: Concept:SSM topic guide with illustrative starter questions. (DOCX 68 kb)

## Abbreviations

NHS: National Health Service
SSM: Support for self-management
