# Supplementary material for: “Was that a success or not a success?”: a qualitative study of health professionals’ perspectives on support for people with long-term conditions
Source: BMC Fam Pract. 2017 Mar 20;18:39. doi: 10.1186/s12875-017-0611-7 (PMC5360072; doi:10.1186/s12875-017-0611-7)
Supplement: Additional file 1: — Concept:SSM topic guide with illustrative starter questions. (DOCX 68 kb) [file 12875_2017_611_MOESM1_ESM.docx]

**
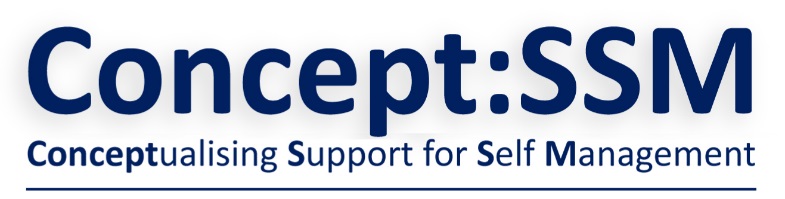
**

**Concept SSM – Topic guide with illustrative question wording**

1. **Opening**

Thank you again for agreeing to speak with me today. As we’ve outlined in our study information leaflet, I’d like to ask you some questions today about your views and experiences of engaging with patients who have long term conditions.

This is for a study that aims to support the development of better training and advice for clinicians working with people with long-term conditions. The interview should take no more than 40 minutes.

[check re. questions before starting]

So, for me to get some background to yourself, I wonder if you could you start by talking a bit about how you came to specialise in the area of practice that you do?

1. **Main**

OK, so I’d like now to talk more specifically about the work you do with people with diabetes/PD. Inevitably, that work will sometimes be very successful, sometimes less so. I’d like to explore this distinction with you a little.

To start with, can I ask for some examples or ‘case histories’ from your experience, to illustrate your idea of success?

And now can I ask for some case histories, again from your own experience, to illustrate the other end of the spectrum? What does unsuccessful look like?

Thinking about the different case histories you’ve given me, how would you define success in you work with people with diabetes/PD?

Do you think your patients would have the same ideas of success as you or not? What happens when they have different ideas?

Why is work with people with diabetes/PD sometimes more successful, why less so? [prompt examples to support this ]

- Are there cases when things turn around (go from unsuccessful to successful)? What happens?
- Are there cases when things fall to pieces (go from successful to unsuccessful)? What happens?

What kinds of things would enable you to achieve ‘success’ more consistently?

- In your view, how does your work environment impact on your ability to successfully work with patients with diabetes/PD?

- Are there any issues that enable/impede you from working in what you consider to be good ways?

There are policy expectations that health professionals involve patients collaboratively in their care. What do you think about that?

- What does it mean to you to work ‘collaboratively’ with patients?

- Do you think it’s something that’s important or not so important – why? [probe views re. means to an end/end in itself?]

- How does that square in your everyday practice?

1. **Closing**

So, to wrap up, I wonder if you could tell me why you agreed to participate in the study?

Is there anything you were hoping to talk about that we haven’t discussed?

Thank you….
